# Identification of an epigenetic signature in human induced pluripotent stem cells using a linear machine learning model

**DOI:** 10.1007/s13577-020-00446-3

**Published:** 2020-10-12

**Authors:** Koichiro Nishino, Ken Takasawa, Kohji Okamura, Yoshikazu Arai, Asato Sekiya, Hidenori Akutsu, Akihiro Umezawa

**Affiliations:** 1 Laboratory of Veterinary Biochemistry and Molecular Biology, Graduate School of Medicine and Veterinary Medicine/Faculty of Agriculture, University of Miyazaki, Miyazaki, Japan; 2 Center for Animal Disease Control, University of Miyazaki, Miyazaki, Japan; 3 Department of Systems BioMedicine, National Research Institute for Child Health and Development, Tokyo, Japan; 4 Department of Reproductive Biology, Center for Regenerative Medicine, National Research Institute for Child Health and Development, Tokyo, Japan

**Keywords:** Machine learning, Human iPSCs, Human ESCs, DNA methylation, Epigenetic signature of hiPSCs

## Abstract

The use of human induced pluripotent stem cells (iPSCs), used as an alternative to human embryonic stem cells (ESCs), is a potential solution to challenges, such as immune rejection, and does not involve the ethical issues concerning the use of ESCs in regenerative medicine, thereby enabling developments in biological research. However, comparative analyses from previous studies have not indicated any specific feature that distinguishes iPSCs from ESCs. Therefore, in this study, we established a linear classification-based learning model to distinguish among ESCs, iPSCs, embryonal carcinoma cells (ECCs), and somatic cells on the basis of their DNA methylation profiles. The highest accuracy achieved by the learned models in identifying the cell type was 94.23%. In addition, the epigenetic signature of iPSCs, which is distinct from that of ESCs, was identified by component analysis of the learned models. The iPSC-specific regions with methylation fluctuations were abundant on chromosomes 7, 8, 12, and 22. The method developed in this study can be utilized with comprehensive data and widely applied to many aspects of molecular biology research.

**Electronic supplementary material:**

The online version of this article (10.1007/s13577-020-00446-3) contains supplementary material, which is available to authorized users.

## Introduction

The application of human induced pluripotent stem cells (iPSCs) in medicine requires prior assessment of the cells with respect to quality, including identity, equivalence, and safety. For evaluation of the iPSCs, comprehensive molecular analysis of characteristics, such as DNA methylation, rather than tests based on a few marker genes, is considered to be more useful. DNA methylation is an epigenetic modification with important roles in normal development and differentiation [1–6]. DNA methylation profiles vary depending on tissue types and cell lineage [5, 7]; therefore, the DNA methylation profile of a cell can be useful for the identification and validation of its cell type. Epigenetic reprogramming, which involves conversion of the DNA methylation profile from somatic to pluripotent cell type, is an essential for the transformation of somatic cells into iPSCs; the cells that acquire the DNA methylation profile of embryonic stem cells (ESCs) become iPSCs [8, 9].

Human iPSCs lower the rate of immune rejection and help in resolving ethical issues associated with the use of ESCs in regenerative medicine [10]. Since the successful development of iPSCs [11–13], comparative analyses between iPSCs and ESCs have been performed by many researchers. Choi et al. [14] reported that there are no molecular or functional differences between genetically matched human ESCs and iPSCs. On the other hand, several studies have identified differentially methylated DNA regions between human iPSCs and ESCs [8, 15–17]. However, these studies only analyzed single point of passage of human iPSCs. In a previous study, we comparatively analyzed several points of passages of 22 human iPSC lines and the results indicated the presence of aberrant hypermethylated sites in iPSCs; however, aberrant hypermethylation in iPSCs occurs stochastically throughout the genome and there is no iPSC-specific aberrant methylated site common to all iPSCs [9]. Despite the lack of DNA methylation hotspots in iPSCs, previous studies have suggested that there are fundamental differences between ESCs and iPSCs, raising questions regarding the extent of similarity between ESC-type epigenome and the reconstructed whole genome of iPSCs. For comparative analysis of cell types with no clear differences, machine learning technology may be useful.

Machine learning is a data analysis technique that attempts to train computers to learn through experience with datasets, in manner similar to natural learning in human. Supervised machine learning can be used to build models for evidence-based prediction, even when there is uncertainly. A supervised learning algorithm trains a machine learning model on a set of input data and the resultant responses (outputs), so that it can reasonably predict the response to new data. In supervised machine learning, classification or regression methods are used to construct predictive models. Classification models are trained to classify the data into categories. Regression models are used to estimate one variable based on the data.

If a model capable of discriminating between ESCs and iPSCs can be constructed using supervised machine learning, the difference between the two cell types could be elucidated. Such a model could help identify the factors underlying the differences between ESCs and iPSCs, as well as enable visualization of these differences, which cannot be distinguished by the naked human eyes.

In this study, we used classification method-based machine learning to create a model that can discriminate between iPSCs and ESCs on the basis of DNA methylation profiles. Further, we attempted to determine the difference between iPSCs and ESCs by analyzing the components of the learning model. Our machine learning-based analysis method and the identified epigenetic indices are useful for evaluating the therapeutic application of human iPSCs. We propose a new method for molecular analysis of the cells that combines comprehensive DNA methylation data and machine learning.

## Materials and methods

### Preparations of mouse embryonic fibroblasts (MEFs) and MEF feeder cells

MEFs were isolated from 13.5-dpc fetuses of pregnant CD1(ICR) mice (Charles River Japan, Inc., Yokohama, Japan) and cultured in Dulbecco’s modified Eagle’s/high-glucose medium (DMEM) (Sigma-Aldrich, St Louis, MO, USA) containing 10% fetal bovine serum (FBS) (Thermo Fisher Scientific, Inc., Waltham, MA, USA, Cat. No. SH3091003), 55 μM 2-mercaptoethanol (Thermo Fisher Scientific), 1% penicillin and streptomycin (Thermo Fisher Scientific). MEFs were irradiated with 30 Gy of gamma irradiation to generate MEF feeder cells. All procedures were performed in accordance with the guidelines for animal care and use of laboratory animals, University of Miyazaki, and the experimental protocols were approved by the Animal Experiment Committee of University of Miyazaki (no. 2012-017, 2017-009).

### Human cell culture

Human endometrium (UtE1104), amnion (AM936EP), placental artery endothelium (PAE551) and menstrual blood (Edom22) cell lines were independently established [18, 19]. Fetal lung fibroblast cells (MRC-5) [20] were obtained from JCRB Cell Bank, Japan. UtE1104, AM936EP, MRC-5, and Edom22 were maintained in POWEREDBY10 medium (Glyco Technica Ltd., Sapporo, Japan). PAE551 were cultured in EGM-2MV BulletKit medium (Lonza, Walkersville, MD, USA) containing 5% FBS (Thermo Fisher Scientific). Human Retro-iPSCs were generated using the retroviral vector pMXs, which contains the cDNAs for human *OCT3/4*, *SOX2*, *c-MYC*, and *KLF4* [8, 9, 19, 21, 22], according to previously described procedures [12] with slight modifications. Episomal-iPSCs were established using the episomal vectors, pCXLE-hOCT3/4-shp53, pCXLE-hSK, and pCXLE-hUL [23], according to previously described procedures [24]. Sendai-iPSCs were produced using the Sendai viral vector SeVdp-iPS, which contains the polycistronic cDNAs for mouse *Oct3/4*, *Sox2*, *c-Myc*, and *Klf4* [23], according to previously described procedures [25]. The SEES lines of human ESCs were generated in the Center for Regenerative Medicine, National Research Institute for Child Health and Development, Tokyo, Japan [26]. Genomic DNA of the HUES lines of human ESCs [27, 28], was kindly gifted by Drs. C. Cowan and T. Tenzan (Harvard Stem Cell Institute, Harvard University, Cambridge, MA, USA). Human iPSCs were maintained on irradiated MEF feeder cells in KnockOut™ Dulbecco’s modified Eagle medium (KO-DMEM) (Thermo Fisher Scientific) containing 20% knockout-serum replacement (Thermo Fisher Scientific), 1% GlutaMAX (Thermo Fisher Scientific), 1% nonessential amino acids (Thermo Fisher Scientific), 55 μM 2-mercaptoethanol (Thermo Fisher Scientific), 1% penicillin and streptomycin (Thermo Fisher Scientific), and 10 ng/ml recombinant human basic fibroblast growth factor (bFGF) (Wako Pure Chemical Industries, Ltd., Osaka, Japan). The human embryonal carcinoma cell lines NCR-G2, NCR-G3 and NCR-G4, which were established in the National Research Institute for Child Health and Development, Tokyo, Japan [29], were cultured in G031101 medium [21]. The human embryonal carcinoma cell lines NCC-IT-A3 [30], PA-1 [31], NEC8, and NEC14 [32] were obtained from JCRB Cell Bank, Japan. NCC-IT-A3, NEC8 and NEC14 were cultured in RPMI1640 medium (Sigma-Aldrich) supplemented with 10% FBS (Thermo Fisher Scientific), and PA-1 was cultured in MEM supplemented with nonessential amino acids and 10% FBS (Thermo Fisher Scientific). The human ECC lines 1777N Rpmet [33] and NTERA-2 [34] were obtained from DS Pharma Biological Co. LTD, Japan, and were cultured in DMEM supplemented with 10% FBS (Thermo Fisher Scientific). All human cell lines used in this study are summarized in Supplemental Table 1.

### DNA methylation analysis

DNA methylation profiles were obtained from each sample using the Illumina Infinium assay with the Infinium HumanMethylation450K BeadChip and Infinium MethylationEPIC BeadChip (Illumina Inc., San Diego, CA, USA). Genomic DNA was extracted from the cells using the QIAamp DNA Mini Kit (Qiagen, Hilden, Germany). From each sample, 1 µg of genomic DNA was subjected to bisulfite conversion using the EZ DNA Methylation kit (Zymo Research, Orange, CA, USA), according to the manufacturer’s recommendations. Following bisulfite conversion, the genomic DNA was hybridized with the Infinium HumanMethylation450K BeadChip and MethylationEPIC BeadChip, and each BeadChip was scanned on an iScan (Illumina Inc.) according to the manufacturer’s instructions. GenomeStudio (Illumina Inc.) was used for background subtraction and normalization of data. Methylated and unmethylated signals were used to compute the *β* value, a quantitative score of the DNA methylation rate that ranges from “0.00”, for completely unmethylated state to “1.00”, for completely methylated state. Additional DNA methylation data were obtained from the NCBI database. Detailed information of cell lines and accession numbers used in this study is mentioned in Supplemental Table 1. Common probes between 450K and EPIC were selected. The probes with sequences that overlapped with variants showing minor allele frequency (MAF) ≥ 5% [35] and detection *p* value ≥ 0.05 (computed from the background based on negative controls) were eliminated from further analysis. A total of 385,683 CpG sites were analyzed in 104 samples including 27 ESC lines, 43 iPSC lines, 9 ECC lines, and 25 somatic cell lines. Unsupervised hierarchical clustering (HCA) with Euclian distance and group average method and principal component analysis (PCA) were used for data analysis. A differentially methylated region (DMR) was characterized by a CpG site having a score that differed by ≥ 0.3 points with respect to the *β* values between two groups. For comparing the average number of DMRs between ESCs and iPSCs, 15 samples were randomly selected from 27 ESC lines and 47 iPSC lines, and the number of DMRs was counted. This step was repeated 100 times and the average number of DMRs was calculated. For comparing the average number of CpG sites within a certain range of standard deviation (SD) between ESCs and iPSCs, 15 samples were randomly selected from 27 ESC lines and 47 iPSC lines, and the number of CpG sites within a certain range of SD was counted. This step was repeated 100 times and the average number of CpG sites within a certain range of SD was calculated.

### Machine learning

Jubatus, a machine learning analytical platform, is an online open-source software (https://jubat.us/en/) developed by Preferred Infrastructure, Inc. (Tokyo, Japan) and NTT SIC (Tokyo, Japan). Multi-class classification (one-vs-others) of the cell types was performed using the classification module Jubaclassifier with Adaptive Regularization of Weight vectors (AROW) [36], which is a linear classification model supported by Jubatus. To perform 4-fold cross-validation, each cell line was divided into four groups, A–D (Supplemental Table 1), and the following four learning series were used: Series-1 comprising training dataset, BCD and test dataset, A; Series-2 comprising training dataset, CDA and test dataset, B; Series-3 comprising training dataset, DAB and test dataset, C; and Series-4 comprising training dataset, ABC and test dataset, D. The training datasets were used for learning in constructing learning models, and test datasets as unknowns were used for validation of the learned models. The construction of learning models was entailed by the random selection of one sample from the training dataset, followed by the input of the DNA methylation rates of 385,683 CpG sites and the cell type of the selected sample into Jubatus followed by learning, thereby updating the learning model. This process was repeated for all the samples in the training dataset, and learning once with all the samples in the training dataset was designated as 1 epoch. In total, 300 epochs were performed and the learned model was assessed every 10 epochs. The adaptive regularization parameter was evaluated using variable regularization weight values of “0.10”, “0.25”, “0.50”, “0.90”, “1.00”, and “1.10”. The learned model was delineated using four classification models corresponding to the cell types (ESCs, iPSCs, ECCs and somatic cells). The source code is available on GitHub (https://github.com/aknishino/20191212_Jub). For evaluating the learned models, Precision, Recall and F-score, Macro-average Precision (Precision_Macro_), Macro-average Recall (Recall_Macro_) and Macro-average F-score (F-score_Macro_) of the each learned model were calculated using the formulae shown in Table 1.

### Sodium bisulfite sequencing

Sodium bisulfite treatment of genomic DNA was carried out using the EZ DNA Methylation-Gold kit (Zymo Research). PCR amplification was performed using BIOTAQ™ HS DNA polymerase (Bioline Ltd, London, UK) with specific primers for *CSMD1*, *FZD10*, *DNAH9*, *FAM19A5*, *TMEM132C*, and *TMEM132D*. The primers used in this study are summarized in Supplemental Table 2. To determine the methylation states of individual CpG sites, the PCR product was gel-extracted and subcloned into Eco RV cut-pBluescriptII vector using NEBuilder HiFi DNA Assembly Master Mix (New England BioLabs, Ipswich, MA, USA), and then sequenced. Methylation sites were visualized and quality control was carried out using the web-based tool QUMA (https://quma.cdb.riken.jp/) [37].

### Accession numbers

NCBI GEO: Infinium HumanMethylation450K BeadChip and Infinium MethylationEPIC BeadChip data obtained in this study have been submitted under the accession number GSE141521. Additional DNA methylation data were obtained from the NCBI database. Accession numbers are given in Supplemental Table 1.

## Results

### Comparison of DNA methylation between ESCs and iPSCs

**Fig. 1:**
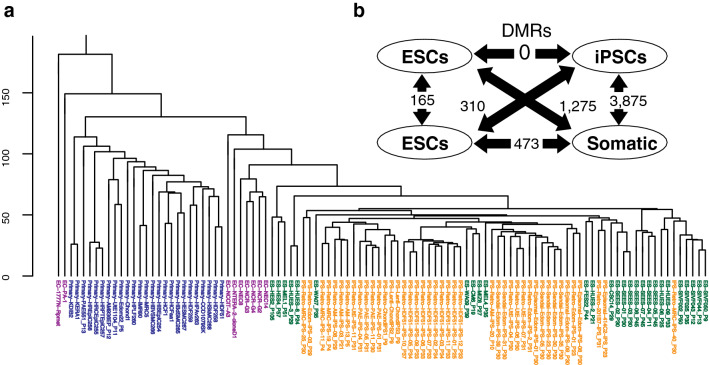
Comparison of DNA methylation between ESCs and iPSCs. **a** Unsupervised HCA based on DNA methylation. Green—ESCs, orange—iPSCs, purple—ECCs and blue—somatic cells. **b** The number of Differentially methylated regions (DMRs) between two types of cell lines

The DNA methylation profiles of 104 human samples, including 27 ESC lines, 43 iPSC lines, 9 ECC lines, and 25 somatic cell lines, were obtained using the Illumina Infinium HumanMethylation array. The methylation rates of 385,683 CpG sites were further analyzed (see details in “Materials and methods”). The promoter regions of the pluripotency-associated genes *POU5F1*, *NANOG*, *SALL4*, *RAB25*, and *EPHA1* showed low levels of methylation, whereas those of the somatic cell-associated genes *GBP3*, *LYST*, and *SP100* were highly methylated in ESC and iPSC lines (Supplemental Fig. 1a). Unsupervised hierarchical cluster analysis (HCA) (Fig. 1a and Supplemental Fig. 1b) and principal component analysis (PCA) (Supplemental Fig. 1c) revealed that iPSCs were clearly distinct from somatic cells and ECCs, but not from ESCs. Comparison between the two types of cells showed that there was no differentially methylated region (DMR) between ESCs and iPSCs (Fig. 1b). These results indicate that there was no clear difference between ESCs and iPSCs.

### Construction of a machine learning model for the classification of cell types

**Fig. 2:**
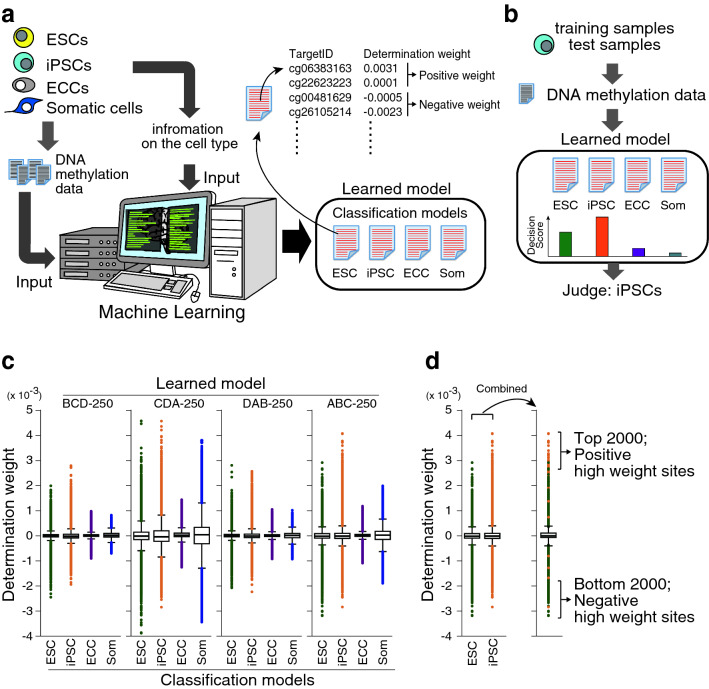
Scheme for machine learning. **a** Constructing a machine learned model. The DNA methylation data and cell type information in the training data set were used as input for the Jubatus classifier program. The four-class classification model was composed of four sets of determination weights corresponding to each cell type. Each classification model comprised a list of determination weights for each CpG sites. TargetID: ID assigned to each CpG site in Illumina HumanMethylation Array. **b** Prediction of the cell type of a sample. The cell type of a sample data was predicted by the learned model using the DNA methylation data. The four-class classification model calculated the integrated quantity obtained by multiplying the DNA methylation rate and the determination weight, and the cell type corresponding to the classification model that produced the largest value was predicted as the cell type of the test data set. For example, if the decision score shows the highest value in iPSC in the learned model, as shown in the figure, the cell type is determined to be iPSC. **c** Boxplots of the determination weight. Dots indicate outliers in each boxplot. **d** Selection of high positive and negative weight sites. The determination files for ESCs and iPSCs in the learned models were combined. Top 2,000 and bottom 2,000 probes were selected as positive and negative high weight sites, respectively

The DNA methylation data of 385,683 CpG sites and information on the cell type of the training samples were used for machine learning (Fig. 2a). In this study, machine learning involved 4-fold cross-validation method, wherein each cell line was divided into four groups to create four datasets (training dataset and test dataset) (Supplemental Table 1) and six different regularization weight values were validated. With each training dataset and regularization weight, 300 epochs were performed; thus, the total number of epochs performed was 7,200 (4 data sets × 6 regularization weight values × 300 epochs). After every 10 epochs, learning results were saved and thus, 720 learning results were obtained as learning models from each dataset. Each of the 720 learning models was used to discriminate the training dataset and the unknowns (test data set) (Fig. 2b), and comparative analyses of the average of the F-score_Macro_ rate were performed (Supplemental Fig. 2b). The learning models from the 250th epoch with AROW regularization weight value of “1.00” had the highest average of the F-score_Macro_ rate from the four models for both training dataset and test dataset, and were therefore selected as the optimal learning models. The highest average of the F-score_Macro_ rate of the test data set, which was achieved by the optimal learning model, was 94.36% (Supplemental Fig. 2a, b). The accuracy, Precision_Macro_, Recall_Macro_, and F-score_Macro_ rates of the test data set in the mixed four models were 94.23%, 95.17%, 93.63% and 94.39%, respectively (Table 1). The accuracy, Precision_Macro_, and Recall_Macro_ rates of the test data set in each four models were shown in Supplemental Fig. 3. The learning model distinguished ESCs from iPSCs with an accuracy of ≥ 81.82% (Supplemental Fig. 3). These results indicated that the learning model generated in the 250th epoch, with a regularization weight value of 1.00, is able to distinguish iPSCs from ESCs with a high efficiency.

**Table 1:** Prediction accuracy, precision, recall and F-score in test samples

Class predicted	ESC	iPSC	ECC	Somatic cells	Precision	Recall
ESCs (*n* = 27)	25 (a1)	2 (b1)	0 (c1)	0 (d1)	89.29% (e1)	92.59% (f1)
iPSCs (*n* = 43)	3 (a2)	40 (b2)	0 (c2)	0 (d2)	95.24% (e2)	93.02% (f2)
ECCs (*n* = 9)	0 (a3)	0 (b3)	8 (c3)	1 (d3)	100.00% (e3)	88.89% (f3)
Somatic cells (*n* = 25)	0 (a4)	0 (b4)	0 (c4)	25 (d4)	96.15% (e4)	100.00% (f4)
Precision (e1) = a1/(a1 + a2 + a3 + a4) Recall (f1) = a1/(a1 + b1 + c1 + d1) Precision (e2) = b2/(b1 + b2 + b3 + b4) Recall (f2) = b2/(a2 + b2 + c2 + d2) Precision (e3) = c3/(c1 + c2 + c3 + c4) Recall (f3) = c3/(a3 + b3 + c3 + d3) Precision (e4) = d4/(d1 + d2 + d3 + d4) Recall (f4) = d4/(a4 + b4 + c4 + d4) Precision_Macro_ = (e1 + e2 + e3 + e4)/4 Recall _Macro_ = (f1 + f2 + f3 + f4)/4 F-score_Macro_ = 2 × Precision_Macro_ × Recall_Macro_/(Precision_Macro_ + Recall_Macro_) Accuracy = (a1 + b2 + c3 + d4)/(27 + 43 + 9 + 25)					Precision_Macro_	Recall_Macro_
					95.17%	93.63%
					Accuracy	F-score_Macro_
					94.23%	94.39%

^a^This prediction was obtained from the learned model at the 250-th epoch with regularization weight “1.00”

### Analysis of components of the learned models

**Fig. 3:**
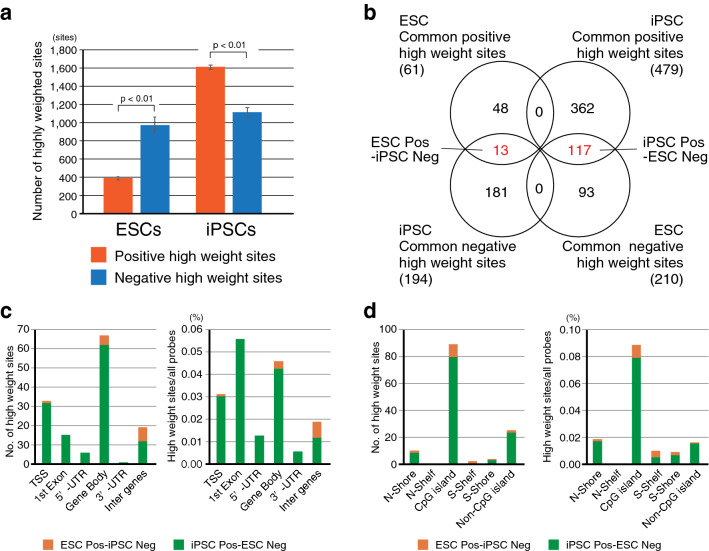
Analysis of determination weights of the learned models. **a** The average number of high weight CpG sites in iPSCs and ESCs. Data are represented as mean ± SEM. **b** Venn-like diagram showing overlapping high weight CpG sites of iPSCs and ESCs. **c** The number (left graph) and proportion (right graph) of the overlapping high weight CpG sites associated with gene figures. **d** The number (left graph) and proportion (right graph) of the overlapping high weight CpG sites associated with CpG islands

Analysis of components of the learned models in the 250th epoch with a regularization weight value of 1.00 was required for better understanding of its capacity to recognize ESCs and iPSCs, as well as distinguish between them. The learned model was delineated by four classification models, each corresponding to one of the following cell types: ESCs, iPSCs, ECCs, and somatic cells; each classification model comprised a list of determination weights for the 385,683 CpG sites (Fig. 2a). Since the determination weights in the various classification models for the same learned model can be compared directly (Fig. 2c), we selected highly weighted CpG sites for each learned model. The classification models for ESCs and iPSCs in each learned model were combined and the top 2,000 highest-weight CpG sites with positive and negative values were selected from four learned models (Fig. 2d). Using comparative analysis, we found that the average number of highly weighted sites was 2.3 times higher in iPSCs than in ESCs. Interestingly, the average number of negative highly weighted sites was higher than that of the positive highly weighted sites in ESCs, whereas the average number of positive highly weighted sites was higher in iPSCs (Fig. 3a). These results suggest that the machine learned model detected more characteristic CpG sites in iPSCs than in ESCs. By extracting common highly weighted sites, 61 and 479 positive high weight sites and 93 and 181 negative high weight sites were identified in ESCs and iPSCs, respectively (Fig. 3b). By comparing the common highly weighted sites in ESCs with those in iPSCs, we found 13 sites common to ESC positive high weight sites and iPSC negative high weight sites (designated as ESC Pos-iPSC Neg), and 117 sites common to iPSC positive high weight sites and ESC negative high weight sites (designated as iPSC Pos-ESC Neg) (Fig. 3b and Supplemental Table 3). The iPSC Pos-ESC Neg sites were found to be abundant around the transcription start site (TSS), first exon and gene body (Fig. 3c), and CpG island (Fig. 3d).

### Distribution of the iPSC Pos-ESC Neg high weight sites on chromosome

**Fig. 4:**
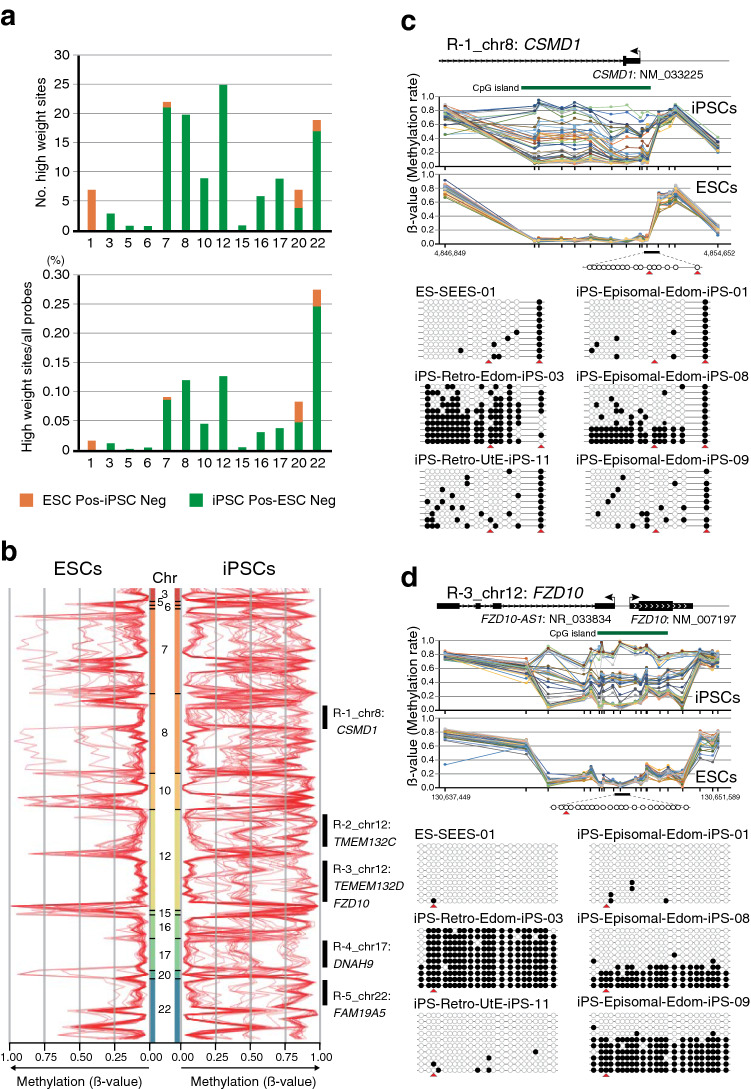
Distribution of the iPSC Pos-ESC Neg high weight sites on chromosomes. **a** The number (upper graph) and proportion (lower graph) of the overlapping high weight CpG sites associated with chromosomes. **b** DNA methylation rate of the iPSC Pos-ESC Neg sites. Five regions (R1–R5) in which the DNA methylation fluctuations were seen only in iPSCs were identified. A red line indicates a cell line. **c**, **d** DNA methylation rate of *CSMD1* (**c**) and *FZD10* (**d**) genes loci and sodium bisulfite sequencing analysis. (Top) Upper and lower graphs shows DNA methylation rates in iPSCs and ESCs, respectively. A line indicates a cell line. (Bottom) Bisulfite sequencing results. Open and closed circles indicate unmethylated and methylated sites, respectively. Red arrowheads represent the position of CpG sites in the Infinium assay. See also Supplemental Fig. 4

The iPSC Pos-ESC Neg sites were found to be abundant on chromosomes 7, 8, 12, and 22 (Fig. 4a). Next, we focused on the DNA methylation rate of high weighted sites. We compared the DNA methylation rates of iPSC Pos-ESC Neg sites in ESCs and iPSCs, and identified five regions in which the DNA methylation fluctuated only in iPSCs (Fig. 4b); these regions were found in the following genes: CUB And Sushi Multiple Domains 1 (*CSMD1*), Transmembrane Protein 132C (*TMEM132C*), Transmembrane Protein 132D (*TMEM132D*), Frizzled 10 (*FZD10*), dynein axonemal heavy chain 9 (*DNAH9*), and TAFA chemokine like family member 5 (*FAM19A5*). The fluctuating regions in these genes were located around the TSS (Fig. 4c, d and Supplemental Fig. 4). To confirm variable methylation at those regions, sodium bisulfite sequencing analysis was performed. Consistent with the results of Infinium HumanMethylation assay, these reasons showed variable methylation in iPSCs (Fig. 4c, d and Supplemental Fig. 4). However, these genes are rarely expressed in ESCs and iPSCs.

### Analysis of the high weight sites

**Fig. 5:**
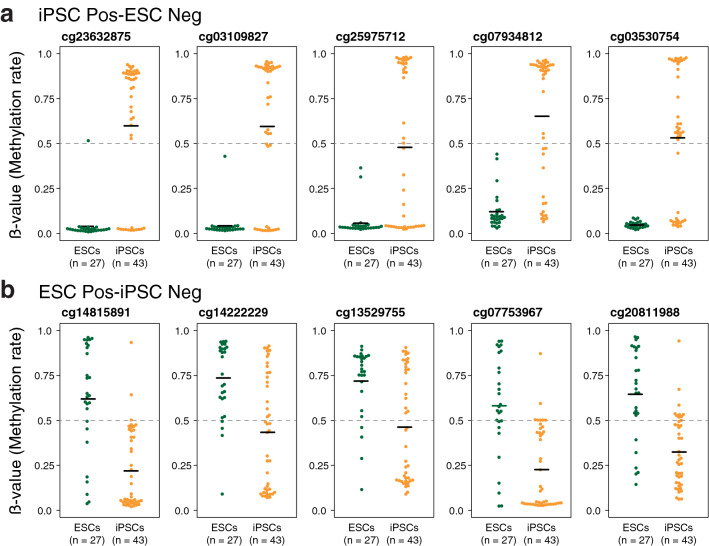
Analysis of the high weight CpG sites. **a** DNA methylation rate of the top five high weight CpG sites in the iPSC Pos-ESC Neg sites. See also Supplemental Fig. 5a. **b** DNA methylation rate of the top five high weight CpG sites in the ESC Pos-iPSC Neg sites. See also Supplemental Fig. 5b

**Fig. 6:**
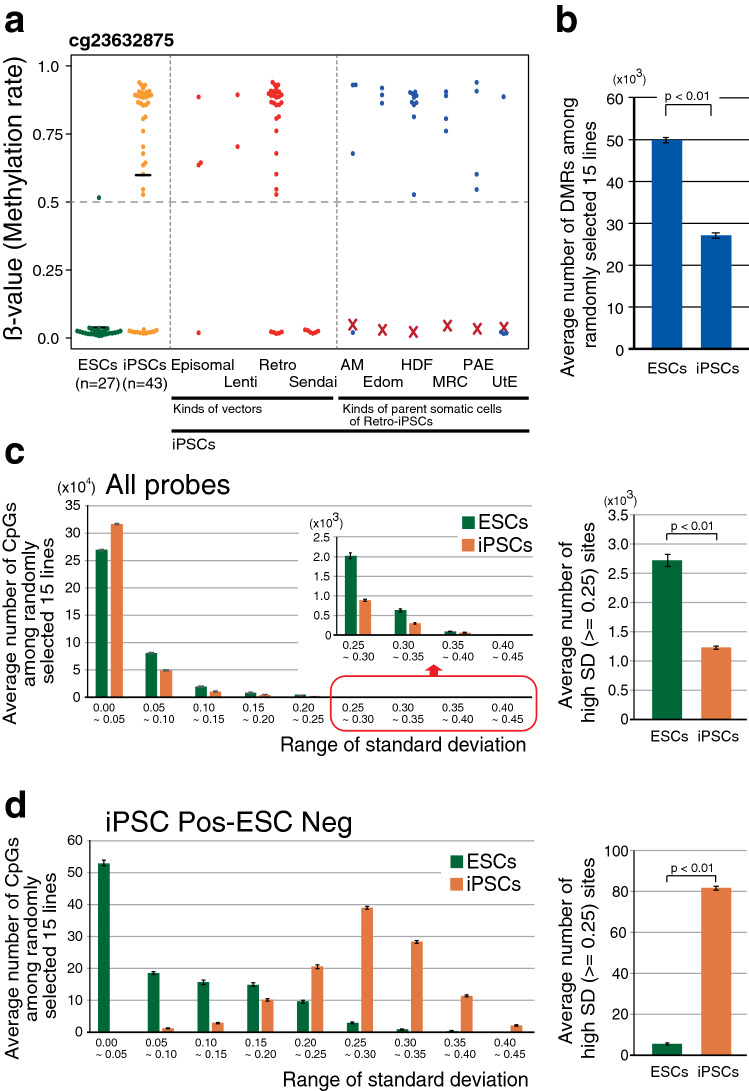
Variations in the DNA methylation rates of the high weight sites. **a** DNA methylation rate of the representative iPSC Pos-ESC Neg sites associated with the methods of iPSC production or type of the parental cells. *X* in the plot indicates the DNA methylation rate of the parental cells. See also Supplemental Fig. 5c. **b** Comparison of the average number of variably methylated regions in the same cell type of ESCs and iPSCs. Data are represented as mean ± SEM. **c**, **d** Average number of CpG sites associated with the range of standard deviation (SD) of DNA methylation rates (left) and the average number of high SD CpG sites (right) in all probes (**c**) and the iPSC Pos-ESC Neg sites (**d**). Data are represented as mean ± SEM

The top ten highly weighted sites were selected and the DNA methylation rates in these sites were compared. The DNA methylation rates of the iPSC Pos-ESC Neg sites in an individual line of iPSCs were found to be widely distributed, whereas ESCs generally had a low methylation rate (Fig. 5a and Supplemental Fig. 5a). On the contrary, the ESC Pos-iPSC Neg sites had varied methylation rates in both ESCs and iPSCs (Fig. 5b and Supplemental Fig. 5b). Variations in the DNA methylation of the high weight sites in iPSCs were not due to the differences in the methods of iPSC generation or the types of the parental cells (Fig. 6a and Supplemental Fig. 5c). Interestingly, the ESCs showed a larger number of variably methylated regions (Fig. 6b) and CpG sites with high standard deviation compared to the iPSCs (Fig. 6c) in the analysis of all CpG sites, indicating that ESCs have more variability in DNA methylation rates than iPSCs. However, in the high weight sites, iPSCs had more CpG sites with high standard deviation compared to ESCs, indicating high variability in iPSCs with respect to methylation levels at the high weight sites (Fig. 6d). These results suggest that the machine learning method was able to determine CpG sites with DNA methylation diversity specific to iPSCs, which can be considered as a characteristic for distinguishing iPSCs from ESCs.

## Discussion

In this study, we developed a new method to distinguish between iPSCs and ESCs on the basis of their DNA methylation profiles. We constructed a learning model based on the linear model for multi-class classification using Jubatus, a machine learning platform. In recent years, deep learning methods have often been used for biological analysis; however, these methods usually require at least 10,000 samples. The availability of only 10–100 variants of human iPSC lines makes the linear model classification system ideal for the analysis of human iPSCs.

iPSCs are essentially an alternative to ESCs, with almost no difference between the two in terms of their properties. iPSC lines generated with non-genome integration methods, such as episomal vector or RNA transfection, are indistinguishable from ESCs in terms of morphology, differentiation ability, gene expression, DNA methylation, etc. [24, 38]. The results obtained in this study are in agreement with previous reports, as no epigenetic features that clearly distinguished iPSCs from ESCs were found. However, our analyses, using a collection of DNA methylation profiles from different types of cells, including 43 iPSC lines which contained Retro-, Lenti-, Sendai-, and Episomal-iPSCs, 27 ESC lines, 9 ECC lines, and 25 somatic cell lines, demonstrated that machine learning with AROW, a linear model for classification, is effective for the discrimination of cell types, especially iPSCs and ESCs. The learned models achieved high-accuracy prediction rates in distinguishing iPSCs from ESCs. In other words, our learned models recognized the differences between iPSCs and ESCs and were able to discriminate between the cell types. Interestingly, the learned models recognized the iPSC lines as iPSCs, irrespective of the production methods used.

One of the advantages of a linear classification-based learning model is the ability to select and analyze components, such as determination weights corresponding to each CpG site. The analysis of the high weight components revealed that the learned models searched for genomic regions that are characteristic of iPSCs and used them to distinguish iPSCs from ESCs. This resulted in the identification of fluctuating iPSC-specific methylation regions, which are especially abundant on chromosomes 7, 8, 12, and 22. DNA methylation was more variable in each of the ESC lines than in iPSCs, indicating that the ESCs possessed more fluctuating methylation regions than the iPSCs. Despite the methylation variation in ESCs, the learned models selected CpG sites with DNA methylation diversity specific to iPSCs as characteristics for distinguishing iPSCs from ESCs. Comparison of the DNA methylation rates of the iPSC Pos-ESC Neg sites led to the identification of fluctuating methylation regions in six genes, including *CSMD1*, *TMEM132C*, *TMEM132D*, *FZD10*, *DNAH9*, and *FAM19A5*. *CSMD1* is known to be a tumor-suppressor gene under the control of DNA methylation in liver cancer and head and neck squamous cell carcinoma [39–41]. *TMEM132C* has been reported to show differential methylation and is downregulated by DNA hypermethylation in breast tumors [42]. *TMEM132D* [43] and *DNAH9* [44] are cancer-associated genes in small cell lung cancer, and *FDZ10* has a role in cancer reactivation [45]. The expression of *FAM19A5*, also known as *TAFA5*, is influenced by the activation of β-catenin [46] and c-Myc promotes the Wnt/β-catenin activity in breast cancers [47]. These genes are involved in carcinogenesis, and the fluctuating regions in these genes are located around the TSS; this suggests that variations in DNA methylation in these genes influence the risk of iPSCs. However, it is seen that these variations in DNA methylation do not affect the gene expression profiles in either ESCs or iPSCs, and also do not exert any influence on pluripotency. Nevertheless, it is possible that the fluctuations in methylation may affect the differentiation properties of iPSCs. The possible effects of such methylation fluctuations on the differentiation properties of iPSCs need to be evaluated through further detailed investigations.

Comparison of iPSCs obtained through different production methods revealed that Sendai-iPSCs were the least diverse in terms of fluctuating methylation regions, and their DNA methylation pattern showed maximum similarity with that of the ESCs. The similarity observed between Sendai-iPSCs and ESCs is consistent with the result of a previously reported comprehensive DNA methylation analysis [23]. However, no significant differences were detected in pluripotency between the Sendai-iPSCs and the iPSCs derived from other production methods [23]. Aberrant DNA methylation at some imprinted gene loci in ESCs and iPSCs has been reported [9, 48, 49], and this abnormality was detected in 68 imprinted genes [23], indicating that aberrant DNA methylation occurs widely in human ESCs and iPSCs. In this study, we identified 130 high weight sites, including 13 ESC Pos-iPSC Neg and 117 iPSC Pos-ESC Neg CpG sites; however, there were no imprinted genes in the 130 high weight sites, suggesting that the abnormalities of imprinted genes are not specific to either iPSCs or ESCs.

In conclusion, we were able to distinguish human iPSCs from ESCs using machine learning methods, even when the cells lacked specific markers. The results of this study will have a significant effect on the use of these cell lines in various in vitro research studies for specific purposes. In addition, an epigenetic signature of iPSCs was identified by component analysis using our learned models. The learned models developed in this study contribute towards enhancing our understanding of the iPSCs at the gene level and hold potential for achieving remarkable advances in various fields of biology research, including computational biology, molecular biology, cell biology, and cancer biology. The approach of the machine learning method used in this study is useful for comprehensive data analysis and can be widely applied to iPSC research as well as many other fields of research in life sciences.

## Electronic supplementary material

Below is the link to the electronic supplementary material.

Supplementary file1 (PDF 957 kb)

Supplementary file2 (XLSX 26 kb)

Supplementary file3 (PDF 76 kb)

Supplementary file4 (XLSX 26 kb)
