# Supplementary material for: Identification of an epigenetic signature in human induced pluripotent stem cells using a linear machine learning model
Source: Hum Cell. 2020 Oct 12;34(1):99–110. doi: 10.1007/s13577-020-00446-3 (PMC7788050; doi:10.1007/s13577-020-00446-3)
Supplement: Supplementary file 1 — Supplementary file1 (PDF 957 kb) [file 13577_2020_446_MOESM1_ESM.pdf]

Supplementary materials

Identification of an epigenetic signature in human induced pluripotent stem cells using a linear machine learning model

Koichiro Nishino[1,2]*, Ken Takasawa[1#a], Kohji Okamura[3] Yoshikazu Arai[1], Asato Sekiya[1], Hidenori Akutsu[4] and Akihiro Umezawa[4]

[1]Laboratory of Veterinary Biochemistry and Molecular Biology, Graduate School of Medicine and Veterinary Medicine/Faculty of Agriculture, University of Miyazaki, Miyazaki, Japan.

[2] Center for Animal Disease Control, University of Miyazaki, Miyazaki, Japan.

[3] Department of Systems BioMedicine, National Research Institute for Child Health and Development, Tokyo, Japan.

[4]Department of Reproductive Biology, Center for Regenerative Medicine, National Research Institute for Child Health and Development, Tokyo, Japan.

[#a] Current address: Division of Molecular Modification and Cancer Biology, National Cancer Center Research Institute, Tokyo, Japan and Cancer Translational Research Team, RIKEN Center for Advanced Intelligence Project, Tokyo, Japan

* Corresponding author

E-mail: aknishino@cc.miyazaki-u.ac.jp (KN)

Tel: +81-985-58-7263

Fax: +81-985-58-7263

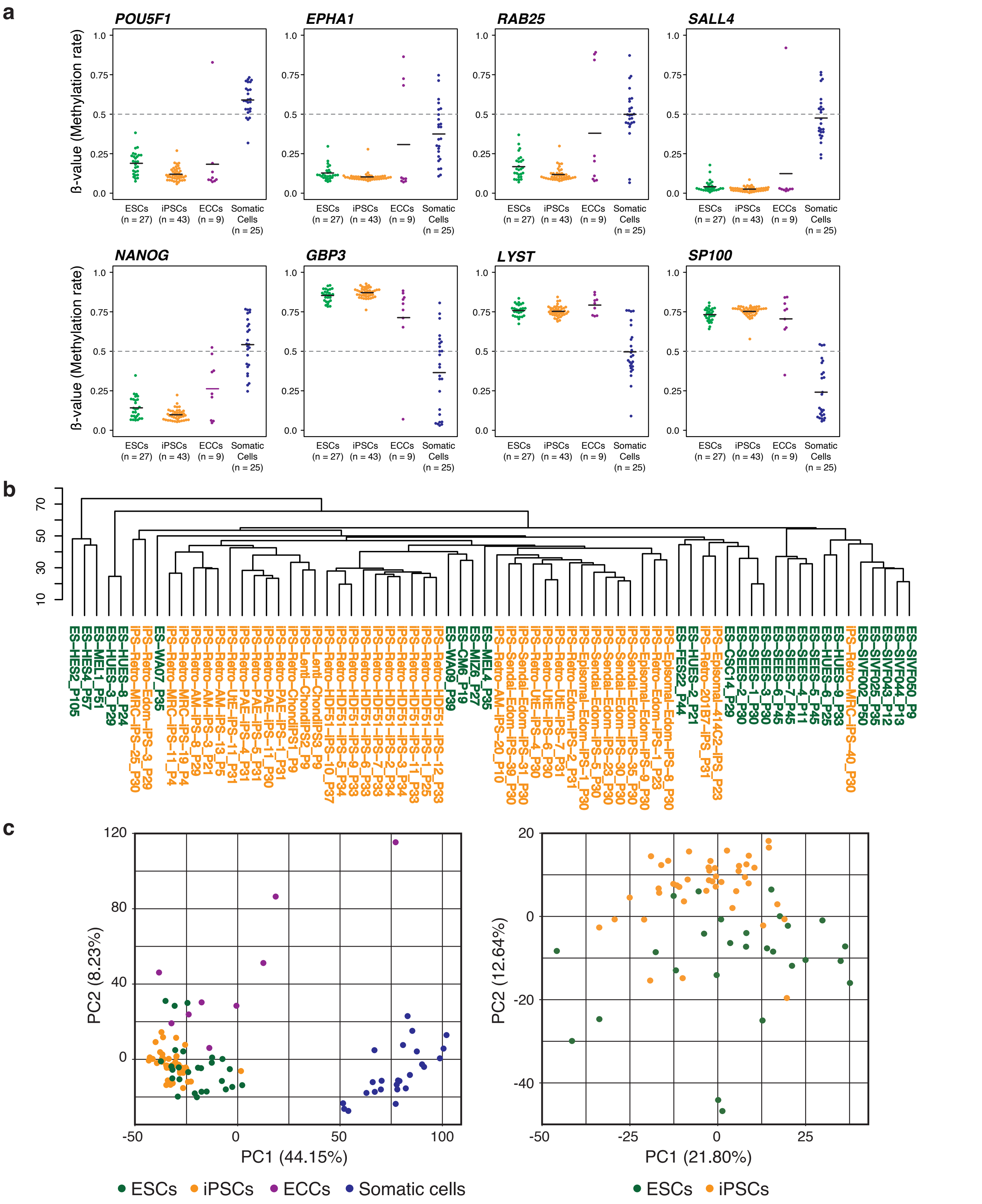

Supplemental Fig. 1 DNA methylation rate at the promoter regions of pluripotency-associated genes and principal component analysis

(a) DNA methylation rate at the promoter regions of *POU5F1*, *EPHA1*, *RAB25*, *SALL4*, *NANOG*, *GBP3*, *LYST* and *SP100*. A plot indicates a cell line. **(b)** Unsupervised HCA based on DNA methylation. Green - ESCs, orange - iPSCs. **(c)** PCA based on DNA methylation. Green - ESCs, orange - iPSCs, purple - ECCs and blue - somatic cells.

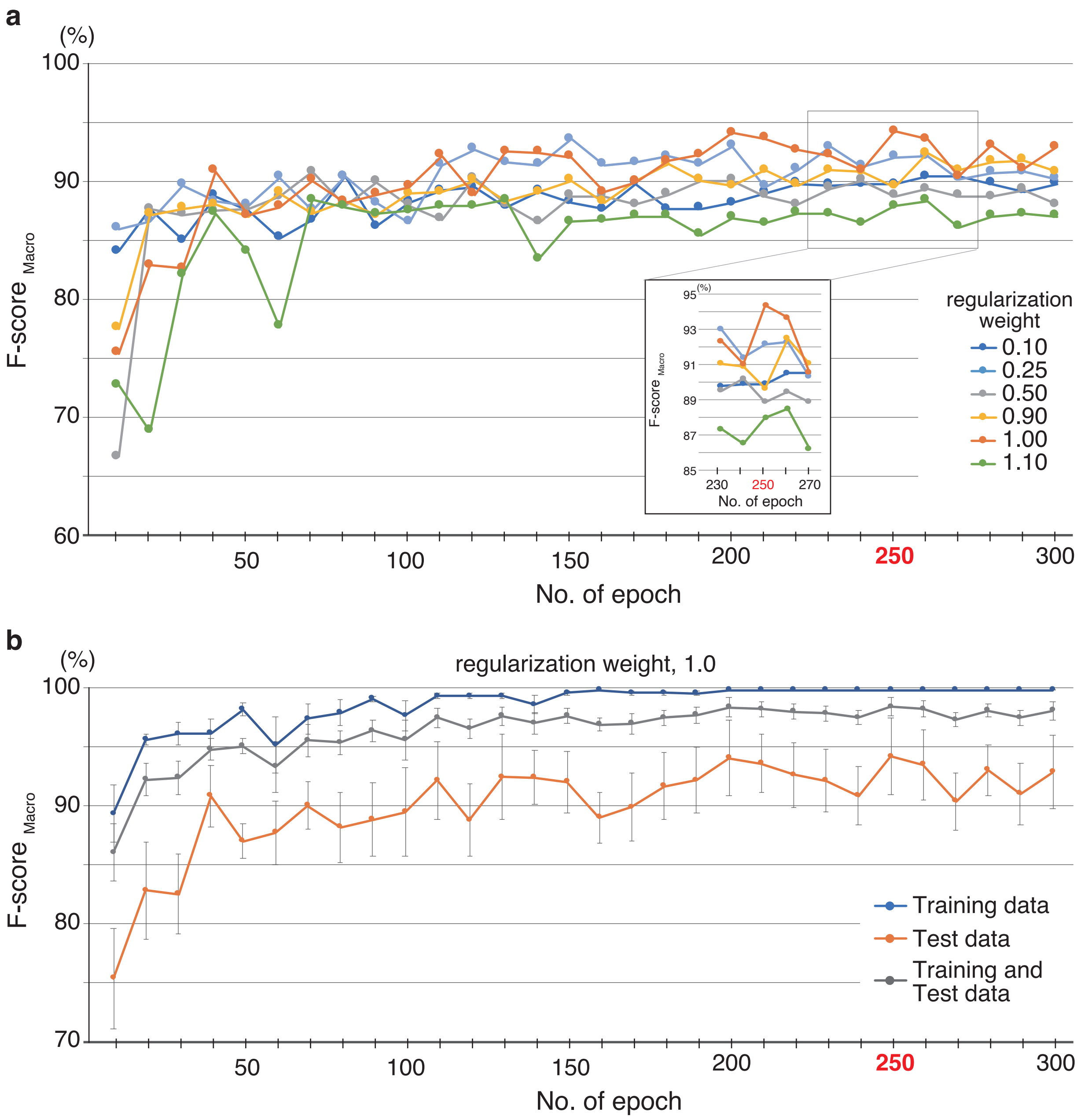

Supplemental Fig. 2 Learning curve obtained from the four learning series

(a) Cross validation of the regularization weight value and epochs in the average of F-score $_{\text{Macro}}$ rate in test data set. The highest average of the F-score $_{\text{Macro}}$ rate was recorded at the 250th epoch with regularization weight value of "1.00" . **(b)** The average of the F-score $_{\text{Macro}}$ rate of the training data set and test data set at the 250th epoch with regularization weight value of "1.00" . The values are shown as the means ± S.E.

Learning Model: BCD-250

Class ＼ Predicted	ESC	iPSC	ECC	Somatic cells
ESCs (n = 7)	6 (a1)	1 (b1)	0 (c1)	0 (d1)
iPSCs (n = 11)	2 (a2)	9 (b2)	0 (c2)	0 (d2)
ECCs (n = 2)	0 (a3)	0 (b3)	1 (c3)	1 (d3)
Somatic cells (n = 6)	0 (a4)	0 (b4)	0 (c4)	6 (d4)

Precision	Recall
75.00% (e1)	85.71% (f1)
90.00% (e2)	81.82% (f2)
100.00% (e3)	50.00% (f3)
85.71% (e4)	100.00% (f4)

Precision $_{Macro}$	Recall $_{Macro}$	F-score $_{Macro}$	Accuracy
87.68%	79.38%	83.32%	84.62%

Learning Model: CDA-250

Class ＼ Predicted	ESC	iPSC	ECC	Somatic cells
ESCs (n = 7)	6 (a1)	1 (b1)	0 (c1)	0 (d1)
iPSCs (n = 11)	0 (a2)	11 (b2)	0 (c2)	0 (d2)
ECCs (n = 2)	0 (a3)	0 (b3)	2 (c3)	0 (d3)
Somatic cells (n = 6)	0 (a4)	0 (b4)	0 (c4)	6 (d4)

Precision	Recall
100.00% (e1)	85.71% (f1)
91.67% (e2)	100.00% (f2)
100.00% (e3)	100.00% (f3)
100.00% (e4)	100.00% (f4)

Precision $_{Macro}$	Recall $_{Macro}$	F-score $_{Macro}$	Accuracy
97.92%	96.43%	97.17%	96.15%

Learning Model: DAB-250

Class ＼ Predicted	ESC	iPSC	ECC	Somatic cells
ESCs (n = 7)	7 (a1)	0 (b1)	0 (c1)	0 (d1)
iPSCs (n = 11)	0 (a2)	11 (b2)	0 (c2)	0 (d2)
ECCs (n = 2)	0 (a3)	0 (b3)	2 (c3)	0 (d3)
Somatic cells (n = 6)	0 (a4)	0 (b4)	0 (c4)	6 (d4)

Precision	Recall
100.00% (e1)	100.00% (f1)
100.00% (e2)	100.00% (f2)
100.00% (e3)	100.00% (f3)
100.00% (e4)	100.00% (f4)

Precision $_{Macro}$	Recall $_{Macro}$	F-score $_{Macro}$	Accuracy
100.00%	100.00%	100.00%	100.00%

Learning Model: ABC-250

Class ＼ Predicted	ESC	iPSC	ECC	Somatic cells
ESCs (n = 6)	6 (a1)	0 (b1)	0 (c1)	0 (d1)
iPSCs (n = 10)	1 (a2)	9 (b2)	0 (c2)	0 (d2)
ECCs (n = 3)	0 (a3)	0 (b3)	3 (c3)	0 (d3)
Somatic cells (n = 7)	0 (a4)	0 (b4)	0 (c4)	7 (d4)

Precision	Recall
85.71% (e1)	100.00% (f1)
100.00% (e2)	90.00% (f2)
100.00% (e3)	100.00% (f3)
100.00% (e4)	100.00% (f4)

Precision $_{Macro}$	Recall $_{Macro}$	F-score $_{Macro}$	Accuracy
96.43%	97.50%	96.96%	96.15%

Average

Precision $_{Macro}$	Recall $_{Macro}$	F-score $_{Macro}$	Accuracy
95.51%	93.33%	94.36%	94.23%

Precision (e1) = a1 / (a1 + a2 + a3 + a4)
Recall (f1) = a1 / (a1 + b1 + c1 + d1)
Precision (e2) = b2 / (b1 + b2 + b3 + b4)
Recall (f2) = b2 / (a2 + b2 +c2 + d2)
Precision (e3) = c3 / (c1 + c2 + c3 + c4)
Recall (f3) = c3 / (a3 + b3 + c3 + d3)
Precision (e4) = d4 / (d1 + d2 + d3 + d4)
Recall (f4) = d4 / (a4 + b4 + c4 + d4)
Precision Macro = (e1 + e2 + e3 + e4) / 4
Recall Macro = (f1 + f2 + f3 + f4) / 4
F-score Macro = 2 • Precision Macro • Recall Macro / (Precision Macro + Recall Macro)
Accuracy = (a1 + b2 + c3 + d4) / 26

Supplemental Fig. 3 The prediction accuracy, F-score $_{Macro}$, Precision $_{Macro}$, and Recall $_{Macro}$ rates of the test data set in each four models at the 250-th epoch with regularization weight "1.00" .

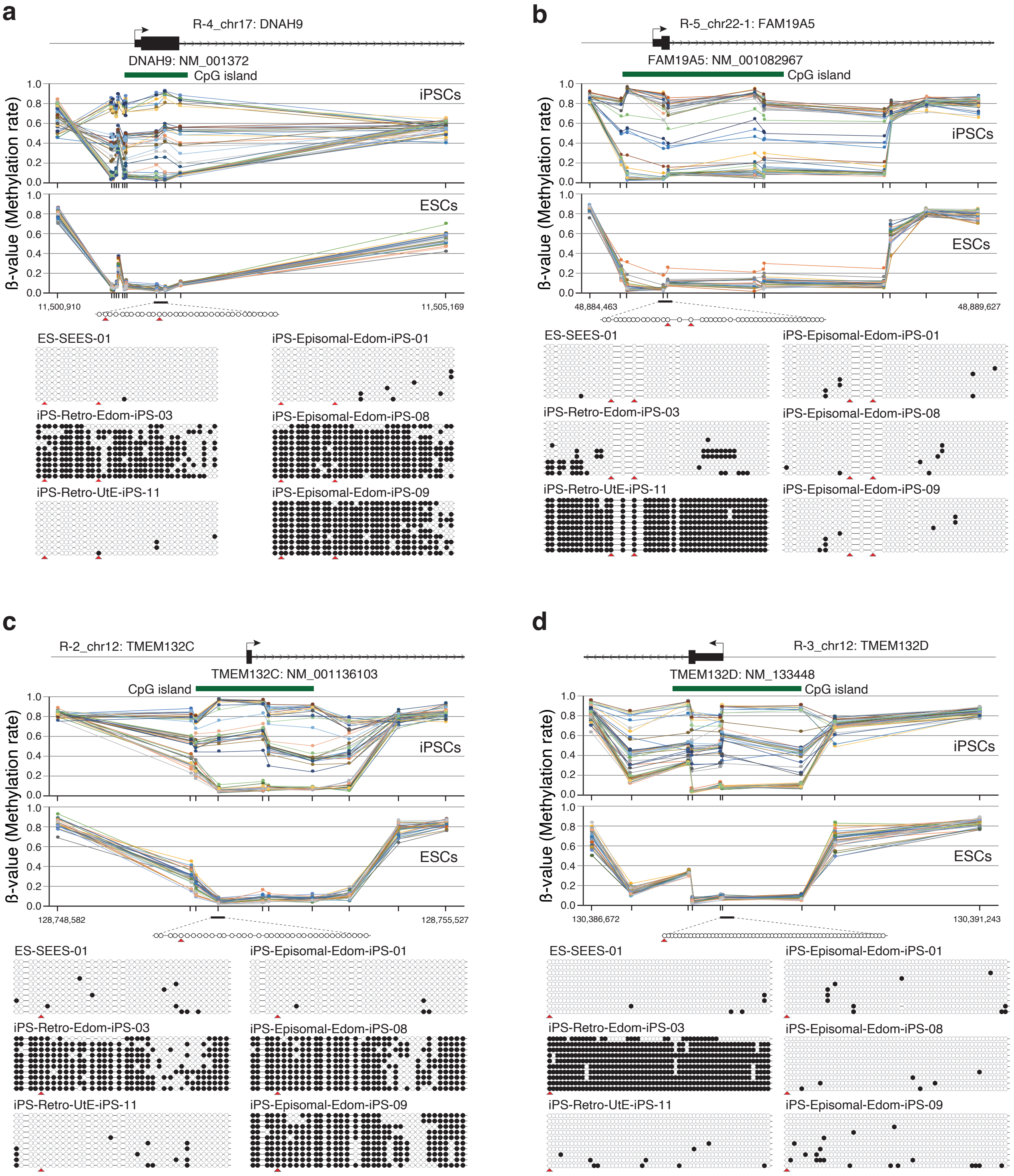

Supplemental Fig. 4 DNA methylation rate of the genes that have the iPSC Pos-ESC Neg sites

DNA methylation rate of *DNAH9* **(a)**, *FAM19A5* **(b)**, *TMEM132C* **(c)**, and *TMEM132D* **(d)** genes loci and sodium bisulfite sequencing analysis. (Top) Upper and lower graphs show DNA methylation rates in iPSCs and ESCs, respectively. A line indicates a cell line. (Bottom) Bisulfite sequencing results. Open and closed circles indicate unmethylated and methylated sites, respectively. Red arrowheads represent the position of CpG sites in the Infinium assay.

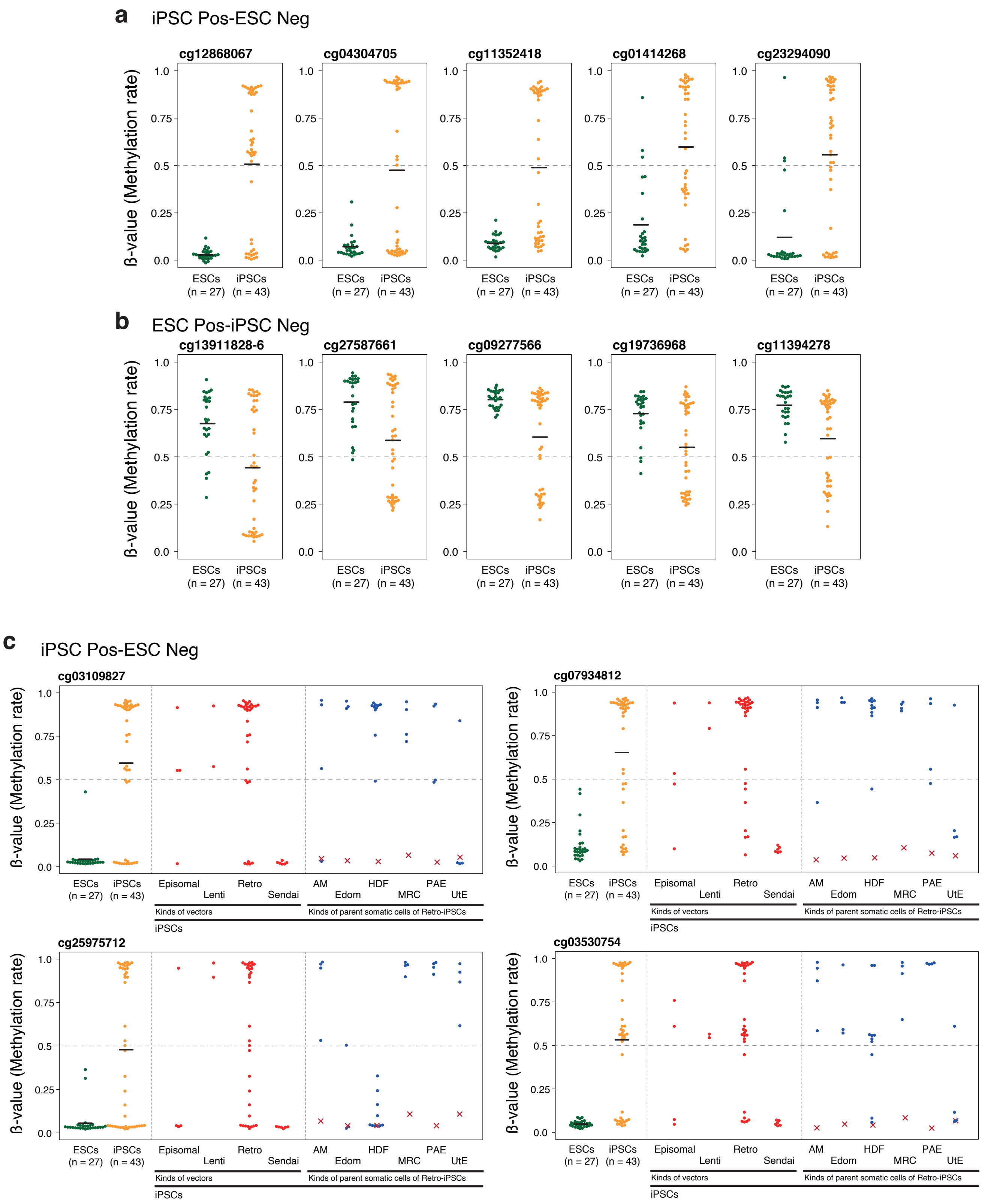

Supplemental Fig. 5 Analysis of the high weight CpG sites

(a) DNA methylation rate of the top 6-10th high weight CpG sites in the iPSC Pos-ESC Neg sites. **(b)** DNA methylation rate of the top 6-10th high weight CpG sites in the ESC Pos-iPSC Neg sites. **(c)** DNA methylation rate of the representative iPSC Pos-ESC Neg sites associated with the methods of iPSC production or type of the parental cells. X in the plot indicates the DNA methylation rate of the parental cells.
