# Supplementary material for: Identification of an epigenetic signature in human induced pluripotent stem cells using a linear machine learning model
Source: Hum Cell. 2020 Oct 12;34(1):99–110. doi: 10.1007/s13577-020-00446-3 (PMC7788050; doi:10.1007/s13577-020-00446-3)
Supplement: Supplementary file 3 — Supplementary file3 (PDF 76 kb) [file 13577_2020_446_MOESM3_ESM.pdf]

Supplemental Table 2. Primers for bisulfite PCR.

ID	Sequences	Size	Annealing temp.
Bis-CSMD1-F	5'- *atcgataagcttgat*TaYggYgTagtTTTagaTagaTagagT-3'		
Bis-CSMD1-R	5'- *ctgcaggaattcgat*gtcttaattctaAaAacttcctcatAtcac-3'	534 bp	55 ˚C
Bis-FZD10-F	5'- *atcgataagcttgat*TaggaagatgYgggTaggtttaTaagT-3'		
Bis- FZD10-R	5'- *ctgcaggaattcgat*gtctcctcRccctctctctAcctAAcctcca-3'	335 bp	55 ˚C
Bis-DNAH9-F	5'- *atcgataagcttgat*TtgggagYgttgYgYggggagtgTtgag-3'		
Bis-DNAH9-R	5'- *ctgcaggaattcgat*gtctatcactccRcctcttAAacctccccctA-3'	452 bp	60 ˚C
Bis-FAM19A5-F	5'- *atcgataagcttgat*gYgYgggTagggTYggTtgTtgaga-3'		
Bis-FAM19A5-R	5'- *ctgcaggaattcgat*gtctcRaAcRcccRAaacccRAAtAAaccctc-3'	399 bp	60 ˚C
Bis-TMEM132C-F	5'-*atcgataagcttgat*TtgtTCgttgggTaTTCgggTaggagtT-3'		
Bis-TMEM132C-R	5'-*ctgcaggaattcgat*gtctcGcGctccGAccGAAacaacttttctc-3'	376 bp	60 ˚C
Bis-TMEM132D-F	5'- *atcgataagcttgat*TtttTTtgtttaTTYgagYgaagagtggT-3'		
Bis-TMEM132D-R	5'- *ctgcaggaattcgat*gtcAAccctcctccRcRcacatctAAcc-3'	488 bp	55 ˚C

* Italic letters in primer sequences represent recombination arm for subcloning.

* Upper cases in primer sequences represent converted nucleotide for bisulfite PCR
